# Circadian Variation of Migraine Attack Onset Affects fMRI Brain Response to Fearful Faces

**DOI:** 10.3389/fnhum.2022.842426

**Published:** 2022-03-09

**Authors:** Daniel Baksa, Edina Szabo, Natalia Kocsel, Attila Galambos, Andrea Edit Edes, Dorottya Pap, Terezia Zsombok, Mate Magyar, Kinga Gecse, Dora Dobos, Lajos Rudolf Kozak, Gyorgy Bagdy, Gyongyi Kokonyei, Gabriella Juhasz

**Affiliations:** ^1^SE-NAP2 Genetic Brain Imaging Migraine Research Group, Hungarian Brain Research Program, Semmelweis University, Budapest, Hungary; ^2^Department of Pharmacodynamics, Faculty of Pharmacy, Semmelweis University, Budapest, Hungary; ^3^Department of Personality and Clinical Psychology, Institute of Psychology, Faculty of Humanities and Social Sciences, Pázmány Péter Catholic University, Budapest, Hungary; ^4^Center for Pain and the Brain (PAIN Research Group), Department of Anesthesiology, Critical Care and Pain Medicine, Boston Children’s Hospital and Harvard Medical School, Boston, MA, United States; ^5^Institute of Psychology, ELTE Eötvös Loránd University, Budapest, Hungary; ^6^János Szentágothai Doctoral School of Neurosciences, Semmelweis University, Budapest, Hungary; ^7^Department of Neuroradiology, Medical Imaging Centre, Semmelweis University, Budapest, Hungary; ^8^NAP-2-SE New Antidepressant Target Research Group, Hungarian Brain Research Program, Semmelweis University, Budapest, Hungary; ^9^MTA-SE Neuropsychopharmacology and Neurochemistry Research Group, Hungarian Academy of Sciences, Semmelweis University, Budapest, Hungary

**Keywords:** pain, headache onset, emotional processing, brain imaging, emotional faces task, circadian rhythm

## Abstract

**Background:**

Previous studies suggested a circadian variation of migraine attack onset, although, with contradictory results – possibly because of the existence of migraine subgroups with different circadian attack onset peaks. Migraine is primarily a brain disorder, and if the diversity in daily distribution of migraine attack onset reflects an important aspect of migraine, it may also associate with interictal brain activity. Our goal was to assess brain activity differences in episodic migraine subgroups who were classified according to their typical circadian peak of attack onset.

**Methods:**

Two fMRI studies were conducted with migraine without aura patients (*n* = 31 in Study 1, *n* = 48 in Study 2). Among them, three subgroups emerged with typical Morning, Evening, and Varying start of attack onset. Whole brain activity was compared between the groups in an implicit emotional processing fMRI task, comparing fearful, sad, and happy facial stimuli to neutral ones.

**Results:**

In both studies, significantly increased neural activation was detected to fearful (but not sad or happy) faces. In Study 1, the Evening start group showed increased activation compared to the Morning start group in regions involved in emotional, self-referential (left posterior cingulate gyrus, right precuneus), pain (including left middle cingulate, left postcentral, left supramarginal gyri, right Rolandic operculum) and sensory (including bilateral superior temporal gyrus, right Heschl’s gyrus) processing. While in Study 2, the Morning start group showed increased activation compared to the Varying start group at a nominally significant level in regions with pain (right precentral gyrus, right supplementary motor area) and sensory processing (bilateral paracentral lobule) functions.

**Conclusion:**

Our fMRI studies suggest that different circadian attack onset peaks are associated with interictal brain activity differences indicating heterogeneity within migraine patients and alterations in sensitivity to threatening fearful stimuli. Circadian variation of migraine attack onset may be an important characteristic to address in future studies and migraine prophylaxis.

## Introduction

Migraine is a serious and debilitating neurological disorder affecting 1.1 billion people worldwide (Safiri et al., 2022). The most frequent type is episodic migraine without aura characterized by recurrent attacks with typically unilateral, pulsating, moderate or severe headache, accompanying nausea or vomiting, photo- and/or phonophobia. Painful migraine attacks represent only a part of a multiphasic disease with various symptoms usually appearing in a timely order during three phases (excluding migraine aura): (1) *premonitory* (or *prodromal*) *phase* (preceding the headache phase by up to 48–72 h) with symptoms including fatigue, irritability, phonophobia, stiff neck, changes in mood, activity, appetite and sleep-waking rhythms; (2) *migraine attack*; and (3) *postdromal phase* (lingering for 24–48 h after the headache) with symptoms similar to prodromal ones (including tiredness, stiff neck, and difficulties in concentration) (Giffin et al., 2003; Goadsby et al., 2017; May, 2017).

At the moment, we cannot exactly understand or predict the onset of a migraine attack. There are known migraine trigger factors, but many of them may overlap with symptoms of an already ongoing premonitory phase (including sleeping problems, hunger or phonophobia) (Goadsby et al., 2017). Some researchers also suggest that migraine attack onset may show a circadian variation. Recent reviews (Baksa et al., 2019; Poulsen et al., 2021) show contradictory results: although, many authors reported an early morning or late night attack onset peak, others also revealed an afternoon peak and a biphasic diurnal cycle of attacks. One study also reported that most of their investigated migraine patients did not show a constant circadian rhythm of attack onset (de Tommaso and Delussi, 2018). Among the emerging theories, a possible hypothalamic dysfunction has been suggested to explain the diurnal distribution of migraine attacks through hypothalamic involvement in pain modulation and circadian rhythmicity (Park et al., 2018). The main circadian oscillator, the suprachiasmatic nucleus also takes place in the hypothalamus (Saper et al., 2005; Gannon et al., 2014) – the probable causal role of the suprachiasmatic nucleus in migraine periodicity has been suggested more than two decades ago (Zurak, 1997).

A possible explanation for the contradictory results regarding the daily distribution of attack onset may be that subgroups with different circadian attack onset peaks exist within migraine patients. Environmental effects may also contribute to differences in attack onset during the day: morning migraine may be induced by lack of sleep (Alstadhaug et al., 2008), while a peak onset in the afternoon may be connected to work- or school-related stress (Alstadhaug et al., 2008; van Oosterhout et al., 2018). Chronotype, defined broadly as individual differences in preference of daily activity and rest periods, may also influence migraine attack onset: early chronotype was related to earlier attack onset, while late chronotype associated with later attack onset (van Oosterhout et al., 2018). A novel study (Im et al., 2019) revealed that migraineurs with a time preference of headache attack were more likely to have an earlier chronotype compared to migraineurs without a preferential attack time; and migraine patients with later chronotype reported higher attack frequency and later preferential attack time – these associations were specific to migraineurs in contrast to participants with tension-type headache.

Migraine is primarily a brain disorder (Goadsby et al., 2017). Imaging studies revealed that the “migraine brain” shows structural and functional alterations in comparison with healthy controls, even between attacks (i.e., interictally). Although, definitive neuroimaging biomarkers of migraine are still lacking (Russo et al., 2019; Skorobogatykh et al., 2019), functional magnetic resonance imaging (fMRI) studies consistently show altered neural processing of sensory (mostly painful and visual) stimuli interictally compared to healthy controls in several regions, including pre- and postcentral gyrus, superior temporal gyrus, middle and anterior cingulate cortex, visual cortex, middle temporal cortex (for a review see Schwedt et al., 2015). Besides these migraine-specific sensory hypersensitivities, emotional factors are also relevant in migraine: emotional stress is commonly reported as a trigger factor for headache (Andress-Rothrock et al., 2010), increased emotionality during the prodrome is among the best predictors for migraineurs for their attack (Giffin et al., 2003), high level of neuroticism (or emotional lability) is a risk factor for migraine (Magyar et al., 2017) and interestingly, emotional abuse during childhood had a stronger association with migraine (even, after controlling for lifetime depression and anxiety) compared to physical and sexual abuse in a study with a nationally representative sample of young adults in the United States (Tietjen et al., 2017). In accordance with these data, fMRI studies confirmed altered cerebral response to emotional stimuli interictally among migraineurs versus healthy controls in areas including superior and middle frontal gyrus, frontal pole, caudate, thalamus, amygdala, posterior cingulate gyrus, precuneus, cerebellum (Wilcox et al., 2016; Wang et al., 2017; Szabó et al., 2019).

If diversity in daily distribution of migraine attack onset reflects an important aspect of migraine, it may also associate with interictal brain activity. Therefore, our goal was to assess brain activity differences in subgroups of episodic migraineurs who were classified according to their typical circadian peak of attack onset. Comparing these subgroups in an implicit emotional processing fMRI task, we expected activity differences between them in regions previously associated with migraine and related to circadian rhythmicity (hypothalamus), sensory (e.g., superior temporal gyrus, middle and anterior cingulate cortex, visual cortex) and emotional processing (e.g., amygdala, middle frontal gyrus, posterior cingulate gyrus).

## Materials and Methods

Data from two fMRI studies with different participants and MRI scanners were included. In the followings, we detail our methods highlighting differences between Study 1 and Study 2. Study 1 was considered as an exploratory study since it is the first investigation that aims to connect circadian variation of migraine attack onset to fMRI brain activation and typical circadian attack onset peak was based on self-reported questionnaire data. While, in Study 2, our main goal was to replicate the results of Study 1 applying a headache diary to capture typical circadian attack onset peak in a more thorough way.

### Participants

Migraineurs without aura were recruited via advertisements in universities, articles and neurological clinics. Episodic migraine without aura was diagnosed by headache specialists according to the International Classification of Headache Disorders-III criteria (Headache Classification Committee of the International Headache Society [IHS], 2018). Our inclusion criteria comprised of (1) right handedness according to the Edinburgh Handedness Inventory (Oldfield, 1971); (2) normal or corrected to normal vision; (3) lack of history of any chronic medical, neurological (except migraine) or psychiatric disorders diagnosed by senior neurologist and psychiatrist researcher colleagues; (4) lack of daily medication use (except oral contraceptives). Selected migraineurs agreed to avoid to take any prophylactic medication for 3 months and any analgesics or migraine attack medication 48 h before the scan sessions. Further details on response rates and exclusion due to technical problems were published earlier (Kocsel et al., 2019; Szabó et al., 2019).

For Study 1, 34 subjects met the inclusion criteria, further exclusion due to missing data resulted in the final sample of 31 patients with migraine without aura (24 females; mean age: 26.97 years, *SD* = 4.83). In Study 2, applying the same inclusion and exclusion criteria 48 participants (43 females; mean age: 27.02 years, *SD* = 6.29) were eligible for our study with non-missing data.

Written informed consent was provided by all participants, in accordance with the Declaration of Helsinki. The studies were approved by the Scientific and Research Ethics Committee of the Medical Research Council (Hungary).

### Self-Report Measures

In Study 1, subgroups were defined based on self-reported *typical circadian attack onset peak* measured with the following question: “Typically, when does your migraine headache start? Please, choose one answer” from the options of (1) “always in the morning,” (2) “rather in the morning,” (3) “in the forenoon,” (4) “in the afternoon,” (5), “rather in the evening,” (6) “always in the evening,” (7) “at night, during sleep (waking up because of it),” (8) “varying,” and (9) “other.” Options number (1), (2), (3), and (7) represent morning or dawn start (collectively the first half of day) and were combined as *Morning start*; and options (4), (5), and (6) capture afternoon or evening start (covering the second half of the day) and were combined under the name of *Evening start*. A similar categorization to assess a circadian pattern of migraine headache start (namely: “usually before noon” and “usually after noon”) was used in a previous study (Shin et al., 2015). Furthermore, a *Varying start* group was defined: based on options (8) and (9) representing migraineurs without a typical circadian attack onset peak.

In Study 2, all participants were asked to fill a paper headache diary to capture *typical circadian attack onset peak*. An inclusion criterion regarding headache diary was at least two reported migraine attacks (as in the study of de Tommaso and Delussi, 2018) separated at least by a 24 h long headache-free period (as in Alstadhaug et al., 2007). Every reported headaches were separately reviewed, and among them, a migraine-type headache was classified in case of showing at least four of the six migraine attack features listed by ICHD-III (Headache Classification Committee of the International Headache Society [IHS], 2018): (1) 4–72 h long duration, (2) unilateral pain, (3) pulsating pain quality, (4) moderate or severe intensity, (5) aggravation by routine physical activity, and (6) any of the concomitant symptoms (nausea or vomiting, photo- and/or phonophobia). In case of use of an acute migraine treatment, we expected the fulfillment of at least three of the six features. For more details about the used headache diary and exact migraine attack criteria, see Supplementary Appendix 1. Using these inclusion criteria, completed headache diaries covered an average time-span of 2.15 months (minimum: 1, maximum: 6, *SD*: 1.08 months) with 255 migraine-type headaches for the 48 participants. Each patient was included to a typical circadian attack onset peak group based on at least 60% of his/her attack occurrence in the two time slots: from 0:00 to 11:59 (Morning start); 12:00–23:59 (Evening start). Varying start group category was used if someone’s attacks were below 60% in any of the two categories.

The following five variables were used for both studies to control possible confounding effects. *Age* and *sex* are known to be related to migraine (Todd et al., 2018; Straube and Andreou, 2019). *Migraine attack frequency per month* was measured by the question: “How many migraine attacks do you have per month?”. Attack frequency represents an important clinical feature of migraine as it was connected to migraine severity and extent of functional changes in the brain (Maniyar and Goadsby, 2013). It is also a reliable variable: previously, it has been reported as a reasonably accurate self-estimated characteristic of migraine (Niere and Jerak, 2004). As stated in Introduction, chronotype and sleeping problems may affect circadian variation of migraine attack onset. *Chronotype* was measured with the following question: “Do you consider yourself as a morning or an evening type of person?” with the options of (1) “definitely morning,” (2) “rather morning,” (3) “rather evening,” (4) “definitely evening,” (5) “I don’t know.” To gain bigger sample sizes, we combined the first two categories (“definitely/rather morning”) and also categories number (3) and (4) (“definitely/rather evening”). *Sleeping problems* was captured in the following way: “Do you have problems falling asleep or waking up in the middle of the night?” with the options of (1) “never or rarely,” (2) “sometimes,” (3) “frequently or usually.”

### Experimental Task

To measure neural activity, an implicit emotional processing fMRI task was implemented. Subjects were shown gray-scale pictures of adult faces expressing *neutral, fearful, sad*, and *happy* emotions. For face stimuli, a standard set of images (Ekman and Friesen, 1976) was presented in block design. Ensuring attention to stimuli, participants were asked to categorize the sex of faces – this implicit strategy was successfully implemented in neuroimaging studies of emotional facial expressions provoking activation mostly in limbic structures and extrastriate cortical regions (Morris et al., 1998; Surguladze et al., 2003; Radua et al., 2010; Anderson et al., 2011).

Pictures of six adult faces (3–3 males and females) with cropped non-facial features were presented on black background. Three 20 s long rest blocks (white fixation cross at the center) separated the three 20 s long blocks of each emotional expression (happy, sad, and fearful) in a pseudo-random order, distributed with twelve neutral blocks. One block contained six faces. During the 8 min long task, the presentation time for each faces was 3000 ms, and for the interstimulus interval 333 and 334 ms.

The task was presented with the E-Prime 2.0 software (Psychology Software Tools, Inc., Pittsburgh, PA, United States). In the MRI scanner, participants in lying position viewed the face stimuli on a screen through a mirror fixated to the head coil. A two-button response device was used by the participants to indicate the sex of the faces: the participants were instructed to finger-press one button with index finger in case of female faces and the other button with thumb in case of male faces. Before the scan, a brief practice session with neutral faces was completed by the participants on a laptop, outside the scanner room. Behavioral data (accuracy and reaction time) were registered. Previously, the task was thoroughly described and successfully used by our research group (Thomas et al., 2011; Arnone et al., 2012; Szabó et al., 2017, 2019; Dobos et al., 2021).

### fMRI Data Acquisition

MRI scans were timed after 3:00 p.m in the late afternoon/early evening hours. Subjects were asked to avoid to eat, smoke and consume caffeine 4 h prior to the examination.

In Study 1, fMRI data acquisition was performed on a 3 T MRI scanner (Achieva 3 T, Philips Medical System) using a BOLD-sensitive T2*-weighted echo-planar imaging sequence (repetition time TR = 2500 ms, echo time TE = 30 ms, field of view FOV = 240 × 240 mm) with 3 × 3 mm in-plane resolution and contiguous 3 mm slices providing whole-brain coverage. A series of high-resolution anatomical images were also acquired during the imaging session using a T1-weighted 3D TFE sequence with 1 × 1 × 1 mm resolution.

In Study 2, a 3.0 T MAGNETOM Prisma Siemens Syngo scanner was used, with the following parameters: TR = 2220 ms, TE = 30 ms, FOV = 222 × 222 mm, with a 3 × 3 × 3 mm resolution. High-resolution anatomical images were acquired similarly with a 1 × 1 × 1 mm resolution, using a 3D MPRAGE sequence.

### Self-Report and Behavioral Data Analysis

Self-report and behavioral data were analyzed with IBM SPSS Statistics 23. In case of scale variables, to measure potential differences between the subgroups, non-parametric tests were used because of the failure of normality: Kruskal–Wallis test and *post hoc* pairwise Mann–Whitney test with a two-tailed *p* < 0.05 threshold.

In case of categorical variables, Freeman–Halton extension of the Fisher exact probability test was performed for two-rows by three-columns and three-rows by three-columns contingency tables at VassarStats website (Lowry, 2021). Similarly, a two-tailed *p* < 0.05 threshold was set.

### fMRI Data Analysis

For imaging data analysis, Statistical Parametrical Mapping (SPM12) software (The Wellcome Centre for Human Neuroimaging, UCL Queen Square Institute of Neurology, London, United Kingdom) was used in Matlab R2016a (Mathworks). Standard preprocessing steps were implemented: (1) realignment of functional images; (2) coregistration of the mean functional image to the structural image; (3) segmentation; (4) normalization to the Montreal Neurological Institute (MNI) space; (5) smoothing with an 8 mm fullwidth-at-half-maximum (FWHM) Gaussian kernel. Artifact Detection Tools (ART) were used to screen for motion outliers with the following deviation thresholds: more than 3 standard deviations for the global signal; and more than 1 mm in case of scan-to-scan motion. Exclusion criteria was: higher than 15% of volumes registered as outliers. Motion outliers were included as regressors with no interest to the fMRI model.

For first-level analysis, a general linear model (GLM) was applied in SPM12 to measure BOLD-responses to emotional facial expressions with three contrasts: *fear-neutral, sad-neutral, happy-neutral* – the same method was previously used in works of Szabó et al. (2017, 2019). The created contrast maps were entered into second-level analysis. To compare the task-related activation in the whole brain between groups with different typical circadian peak of attack onset, we used one-way ANOVA with five covariates: age, sex, migraine attack frequency per month, chronotype, and sleeping problems. To determine the effect directions between the subgroups with different typical circadian peak of attack onset, *post hoc* pairwise two-sample *t*-tests were implemented with the same five covariates. All fMRI data analyses were performed with an initial threshold of *p* < 0.001 (uncorrected) with a cluster size of *k* ≥ 10 voxels. To adjust for multiple testing, results with a cluster level family-wise error corrected threshold of *p*_FWE_ < 0.05 were considered as statistically significant. Significantly activated clusters were identified with the Automated Anatomical Labeling atlas (aal) (Tzourio-Mazoyer et al., 2002). For visualization of statistical maps, the MNI 152 template brain in MRIcroGL was used.

## Results

### Results of Study 1

#### Self-Reported and Behavioral Results

Answers to the question of *typical circadian attack onset peak* resulted in three subgroups: (1) Morning start (*n* = 8), (2) Evening start (*n* = 9), (3) Varying start (*n* = 14); (nobody selected the options of “in the forenoon” or “other”).

Self-reported characteristics of the Study 1 sample and the subsamples with different typical circadian peak of attack onset, which will be referred as *M*_circ_ subgroups in the manuscript, are collected in Table 1. There was a significant difference in age between the *M*_circ_ subgroups: the Varying start group was older than the Evening start group. No other significant differences were found between the *M*_circ_ subgroups regarding other self-reported data (sex, attack frequency per month, chronotype, sleeping problems).

**TABLE 1:** Details of the Study 1 sample and statistical results of the comparison between *M*_circ_ subgroups.

	Total	Morning start (M)	Evening start (E)	Varying start (V)	Group comparisons
**Participant number (*n*)**	31	8	9	14	
**Sex (*n*, %)**					
*Female*	24 (77.4%)	7 (87.5%)	6 (66.6%)	11 (78.6%)	Fisher’s exact *p* = 0.655
*Male*	7 (22.6%)	1 (12.5%)	3 (33.3%)	3 (21.4%)	
**Age (mean, SD)**	26.97 (4.83)	26.12 (4.32)	23.67 (2.0)	29.57 (5.1)	*H* = 7.516, *p* = 0.023* (V > E; *U* = 21, *p* < 0.008)*
**Attack frequency per month (mean, SD)**	3.34 (3.15)	2.31 (1.13)	4.55 (4.44)	3.14 (2.88)	*H* = 0.139, *p* = 0.933
**Chronotype (*n*, %)**					
*Definitely/rather morning*	13 (41.9%)	4 (50.0%)	2 (22.2%)	7 (50.0%)	Fisher’s exact *p* = 0.223
*Definitely/rather evening*	17 (54.8%)	3 (37.5%)	7 (77.8%)	7 (50.0%)	
*Do not know*	1 (3.2%)	1 (12.5%)	0 (0%)	0 (0%)	
**Sleeping problems (*n*, %)**					
*Never/rarely*	14 (45.2%)	4 (50%)	4 (44.4%)	6 (42.9%)	Fisher’s exact *p* = 0.953
*Sometimes*	14 (45.2%)	4 (50%)	4 (44.4%)	6 (42.9%)	
*Often/usually*	3 (9.7%)	0 (0%)	1 (11.1%)	2 (14.3%)	

H, Kruskal–Wallis test statistic; SD, standard deviation; U, Mann–Whitney test statistic; *, significant effect; M_circ_ subgroups: M, Morning start; E, Evening start; V, Varying start.

Behavioral results of Study 1 are summarized in Supplementary Table 1. No differences were found between the *M*_circ_ subgroups in reaction time and accuracy. Comparing behavioral data in response to different emotions in the total sample, significant differences were detected in reaction time: fear evoked higher reaction time in comparison with happy and neutral faces (for details see Supplementary Table 1).

#### fMRI Results

Main effect of task processing different emotions is summarized in Supplementary Table 4.

#### Group Differences in Brain Response to Emotional Faces

Comparison of whole-brain activation between the three subgroups with different typical circadian peak of attack onset, controlling for five covariates (age, sex, migraine attack frequency per month, sleeping problems, chronotype) resulted in significant differences only in response to fearful faces in one cluster. The cluster included regions of left superior temporal gyrus and left supramarginal gyrus (for details see Table 2). There was no significant difference between the groups in neural response to sad and happy faces.

**TABLE 2:** Brain regions with significant activation differences responding to fearful faces comparing the three *M*_circ_ subgroups in Study 1.

Contrast	Cluster size	Cluster *p* (FWE)	Region	Coordinates (MNI)			Peak *F*-value
				*x*	*y*	*z*	
Fear-neutral	51	0.013	L superior temporal gyrus	–57	–37	17	16.61
			L superior temporal gyrus	–45	–37	20	14.54
			L superior temporal gyrus	–51	–40	20	13.15
			L supramarginal gyrus	–63	–34	23	11.04

*Cluster p (FWE), cluster level family-wise error corrected p-value; L, Left hemisphere; MNI, coordinates in Montreal Neurological Institute (MNI) space; Peak F-value, peak test-statistic of one-way ANOVA.*

*Covariates in the analysis: age, sex, migraine attack frequency per month, sleeping problems, chronotype.*

#### *Post hoc* Pairwise Group Comparisons in Neural Response to Fearful Faces

**FIGURE 1:**
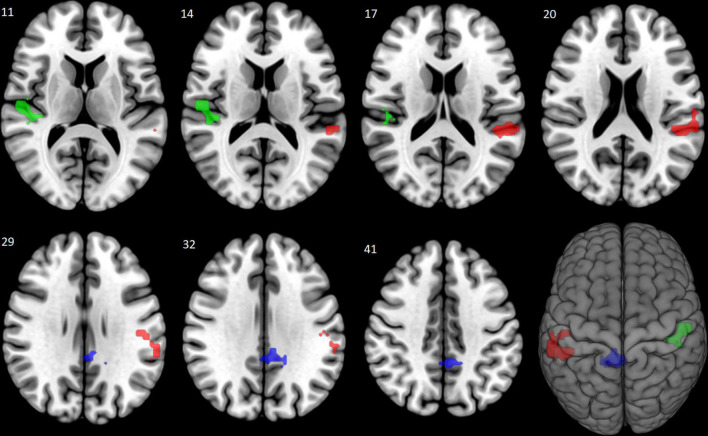
Increased brain activation to fearful faces: Evening start > Morning start (Study 1). The Evening start *M*_circ_ subgroup showed increased brain activation compared to the Morning start subgroup in response to fearful faces. The significantly activated three clusters are shown (in corresponding order shown in Table 3) with red (left superior temporal, left supramarginal and left postcentral gyri), green (right superior temporal gyrus, right Rolandic operculum and right Heschl’s gyrus), and blue (left middle and left posterior cingulate gyri, right precuneus) colors at a cluster level *p*_FWE_ < 0.05, corrected for multiple comparison.

Pairwise group comparisons of the fear-neutral contrast revealed significantly increased brain activation in the Evening start *M*_circ_ subgroup compared to the Morning start subgroup. Three clusters of increased activation were found covering regions of left and right superior temporal gyrus, left supramarginal gyrus, left postcentral gyrus, right Rolandic operculum, right Heschl’s gyrus, left middle cingulate gyrus, left posterior cingulate gyrus and right precuneus (see Table 3 and Figure 1). No other pairwise group comparisons resulted in significant difference.

**TABLE 3:** Brain regions with significantly increased activation responding to fearful faces: Evening start > Morning start (Study 1).

Contrast	Group comparison	Cluster size	Cluster *p* (FWE)	Region	Coordinates (MNI)			Peak *t*-value
					*x*	*y*	*z*	
Fear-neutral	Evening > Morning	132	<0.001	L superior temporal gyrus	–45	–37	20	5.27
				L superior temporal gyrus	–57	–34	17	4.92
				L supramarginal gyrus	–60	–31	23	4.42
				L postcentral gyrus	–51	–22	29	4.26
				L supramarginal gyrus	–60	–25	23	4.19
				L supramarginal gyrus	–57	–31	32	4.03
				L supramarginal gyrus	–60	–40	29	3.75
		63	0.022	R Rolandic operculum	54	–19	11	4.59
				R Heschl’s gyrus	45	–25	14	4.37
				R superior temporal gyrus	42	–28	11	4.08
				R superior temporal gyrus	48	–31	14	3.99
		71	0.013	L middle cingulate gyrus	–9	–40	35	4.36
				L posterior cingulate gyrus	–9	–34	32	4.32
				L middle cingulate gyrus	–15	–46	35	4.26
				L posterior cingulate gyrus	–18	–43	32	4.09
				L middle cingulate gyrus	–12	–43	38	4.06
				L posterior cingulate gyrus	–6	–43	32	4.02
				R precuneus	3	–46	41	3.73

*Cluster p (FWE), cluster level family-wise error corrected p-value; L, Left hemisphere; R, right hemisphere; MNI, coordinates in Montreal Neurological Institute (MNI) space; Peak t-value, peak test-statistic of the two-sample t-test.*

*Covariates in the analysis: age, sex, migraine attack frequency per month, sleeping problems, chronotype.*

### Results of Study 2

#### Self-Reported and Behavioral Results

Regarding *typical circadian attack onset peak*, the same three *M*_circ_ subgroups were detected: (1) Morning start (*n* = 13), (2) Evening start (*n* = 26), and (3) Varying start (*n* = 9).

Self-reported characteristics of the Study 2 sample and the three *M*_circ_ subgroups are presented in Table 4. Again, a significant difference in age was shown between the *M*_circ_ subgroups: the Morning start group was older than the other two groups. No other significant differences were found between the *M*_circ_ subgroups regarding other self-reported data (sex, attack frequency per month, chronotype, sleeping problems).

**TABLE 4:** Details of the Study 2 sample and statistical results of the comparison between *M*_circ_ subsamples with different typical circadian peak of attack onset.

	Total	Morning start (M)	Evening start (E)	Varying start (V)	Group comparisons
**Participant number (*n*)**	48	13	26	9	
**Sex (*n*, %)**					
*Female*	43 (89.6%)	11 (84.6%)	24 (92.3%)	8 (88.9%)	Fisher’s exact *p* = 0.822
*Male*	5 (10.4%)	2 (15.4%)	2 (7.7%)	1 (11.1%)	
**Age (mean, SD)**	27.02 (6.29)	31.23 (7.81)	25.62 (5.2)	25 (4.09)	*H* = 7.354, *p* = 0.025^*^ (M > E; *U* = 86, *p* = 0.013^*^; M > V; *U* = 25, *p* = 0.025^*^)
**Attack frequency per month (mean, SD)**	3.06 (2.68)	2.77 (2.1)	3.02 (2.73)	3.62 (3.46)	*H* = 0.021, *p* = 0.99
**Chronotype (*n*, %)**					
*Definitely/rather morning*	18 (37.5%)	6 (46.2%)	7 (26.9%)	5 (55.6%)	Fisher’s exact *p* = 0.474
*Definitely/rather evening*	28 (58.3%)	7 (53.8%)	17 (65.4%)	4 (44.4%)	
*Do not know*	2 (4.2%)	0 (0%)	2 (7.7%)	0 (0%)	
**Sleeping problems (*n*, %)**					
*Never/rarely*	26 (54.2%)	6 (46.2%)	17 (65.4%)	3 (33.3%)	Fisher’s exact *p* = 0.342
*Sometimes*	16 (33.3%)	6 (46.2%)	6 (23.1%)	4 (44.4%)	
*Often/usually*	6 (12.5%)	1 (7.7%)	3 (11.5%)	2 (22.2%)	
**Headache diary duration (months) (mean, SD)**	2.15 (1.08)	2.23 (0.8)	2.12 (1.2)	2.11 (1.14)	*H* = 0.773, *p* = 0.68

H, Kruskal–Wallis test statistic; SD, standard deviation; U, Mann–Whitney test statistic; *, significant.

Self-reported data was also compared between total samples and *M*_circ_ subgroups of Study 1 and Study 2. The Varying start group was a bit older in Study 1 sample compared to Study 2 sample. Furthermore, distribution of the *M*_circ_ subgroups significantly differed between the two studies: the Morning start and the Evening start group showed higher participant number in Study 2, while the Varying start group had higher participant number in Study 1. For details, see Supplementary Table 3.

Behavioral results of Study 2 are summarized in Supplementary Table 2. Significant differences were found in accuracy between the *M*_circ_ subgroups: the Evening start group processed sad faces with higher accuracy compared to the other two groups, and also neutral faces compared to the Varying start group. Comparing behavioral data in response to different emotions in the total sample, significant differences were detected. Fearful, happy and neutral faces evoked higher accuracy in comparison with sad faces. Furthermore, sad faces associated with higher reaction time compared to happy and neutral faces, and also fearful faces compared to neutral ones. For all details see Supplementary Table 2.

#### fMRI Results

Main effect of task processing different emotions is summarized in Supplementary Table 5.

#### Group Differences in Brain Response to Fearful Faces

Our main goal was to replicate the primary result of Study 1, namely: increased brain activation in response to fearful faces in the Evening start *M*_circ_ subgroup compared to the Morning start subgroup.

Whole-brain activation with the same five covariates as in Study 1 (age, sex, migraine attack frequency per month, sleeping problems, chronotype) was compared between the three *M*_circ_ groups. The ANOVA showed no significant differences between the three groups, however, considering the notably unequal participant number distributions between the *M*_circ_ groups in Study 2, we decided to also run pairwise group comparisons. Among these analyses, only one nominally significant result was found: in response to fearful faces, the Morning start group showed increased brain activation compared to the Varying start group in one cluster covering regions of bilateral paracentral lobule, right precentral gyrus and right supplementary motor area (see Table 5 and Supplementary Figure 1). However, this result does not survive correction for multiple comparison (in case of six *t*-tests: *p* = 0.05/6 = 0.008).

**TABLE 5:** Brain regions with nominally significantly increased activation responding to fearful faces: Morning start > Varying start (Study 2).

Contrast	Group comparison	Cluster size	Cluster *p* (FWE)	Region	Coordinates (MNI)			Peak *t*-value
					*x*	*y*	*z*	
Fear-neutral	Morning > Varying	97	0.012	R paracentral lobule	6	–34	65	4.32
				R precentral gyrus	18	–31	74	4.2
				R supplementary motor area	9	–19	62	3.82
				L paracentral lobule	–3	–37	68	3.69

*Cluster p (FWE), cluster level family-wise error corrected p-value; L, Left hemisphere; R, right hemisphere; MNI, coordinates in Montreal Neurological Institute (MNI) space; Peak t-value, peak test-statistic of the two-sample t-test. Covariates in the analysis: age, sex, migraine attack frequency per month, sleeping problems, chronotype.*

No other significant differences were found between the groups in neural response to sad or happy faces.

## Discussion

Two fMRI studies were conducted to reveal differences in interictal brain activation in an implicit emotional face processing fMRI task as a function of circadian peak of attack onset. In Study 1, later typical circadian attack onset peak was related to significantly increased activation in many brain regions in response to fearful faces in comparison with earlier typical circadian attack onset peak. In Study 2, similarly only fearful (and not happy or sad) faces evoked brain activation differences. However, in this case, higher activation associated with earlier typical circadian attack onset peak compared to varying attack onset peak, and only at a nominal significance level. This is the first investigation connecting circadian variation of migraine attack onset to fMRI brain activation. There may be some important differences between the two studies, mostly the method to capture typical circadian attack onset and the use of different MRI scanners. We will discuss the potential effects of these factors later on. Before that, we would like to highlight that despite the significant methodological differences between the two studies, there are still some overlaps between the results. Although, we have to note that results from Study 2 were significant only at a nominal level, so these should be interpreted with caution.

### Emergence of Migraine Subgroups With Different Typical Circadian Peak of Attack Onset

Our results suggest that subgroups with different typical circadian attack onset peaks may exist within migraine patients. We were able to detect all three predefined *M*_circ_ subgroups in both studies: a Morning start, an Evening start, and a Varying start subgroup. Distribution of the *M*_circ_ subgroups significantly differed between the two studies. In Study 1, using a self-reported question, 45.16% reported Varying start, 29.03% Evening start, and 25.8% Morning start. In Study 2, where a headache diary was used, the Evening start group represented 54.16% of the sample, the Morning start group 27.08% and the Varying start group 18.75%. Altogether, the Evening start group had the highest participant number covering 44.3% of the two samples, while the other two groups showed similar distributions: 29.1% with Varying start and 26.6% with Morning start. Based on these results, even with a broader definition, Evening start (representing the second half of the day) was much more frequent than Morning start (covering the first half of the day). This result might sound surprising because most of the studies conclude that the morning migraine attack start is the most frequent one, however, recent reviews show a much more mixed picture of the field (Baksa et al., 2019; Poulsen et al., 2021). Furthermore, almost one third of the two samples (and nearly half of Study 1 sample) did not report a typical circadian attack onset peak (i.e., Varying start) – this group also needs to be taken into account. In a previous study with episodic and chronic migraineurs, almost 60% did not report a typical diurnal attack onset peak (de Tommaso and Delussi, 2018).

### Overlaps Between fMRI Results of Study 1 and Study 2

First, the Morning start subgroup is involved in both results. In Study 1, this group showed lower neural activation compared to the Evening start group, while in Study 2, higher activation compared to the Varying start group. Thus, we were not able to replicate the results of Study 1 in the same direction, however, these results are not necessarily opposing. A main question about circadian phenomena in migraine: are they related to biological and/or environmental factors? Previously, it was suggested that stress- and sleep-related effects might be more determining in diurnal patterns of migraine attacks than the actual biological clock mechanism (Alstadhaug et al., 2008). We still do not have an answer to this question. At least, the results presented here suggest brain activity differences between migraine subgroups with different typical circadian attack onset peaks.

Second, activity differences between the three groups were found in brain regions with similar functions. Specifically, areas of pain processing, including for example middle cingulate cortex (MCC), postcentral gyrus from Study 1 and precentral gyrus, supplementary motor area (SMA) from Study 2; and regions of sensory processing, including Heschl’s gyrus, precuneus from Study 1 and paracentral lobule from Study 2 were detected. These regions are thought to contribute to migraine attacks (for a review see Schwedt et al., 2015), and some of them also associated with circadian rhythm-related phenomena in previous fMRI studies, including precuneus (Kyeong et al., 2017; Facer-Childs et al., 2019), postcentral gyrus (Kyeong et al., 2017; Fafrowicz et al., 2019), precentral gyrus (Kyeong et al., 2017), posterior cingulate cortex (PCC) (Kyeong et al., 2017), and MCC (Wu et al., 2021).

Third, only fearful (but not happy or sad) faces evoked significant differences in brain activation between the three subgroups – again, suggesting a similar phenomenon detected in Studies 1 and 2. Similarly to pain, fear is also an aversive stimuli and they often co-occur suggesting a strong relation (Vowles et al., 2006) which may be supported by a core aversion-related brain circuit that is commonly responsible for processing painful and non-painful aversive stimuli (Hayes and Northoff, 2011) involving regions overlapping with our identified areas including MCC, PCC (both from Study 1) and SMA (Study 2).

To put our results into broader perspective, next, we discuss them in light of previous emotional processing fMRI studies in migraine.

### Emotional Processing in Migraine

Two previous fMRI studies on processing of emotional stimuli in adult migraineurs compared to healthy controls showed enhanced response selectively to negative (and not positive) emotional stimuli among migraineurs in interictal state (Wilcox et al., 2016; Wang et al., 2017). Increased neural activation was found in regions of superior and middle frontal gyrus, frontal medial cortex, frontal pole, PCC, precuneus, cuneal cortex, caudate, thalamus, left amygdala, right hippocampus, brainstem, and cerebellum in the study of Wilcox et al. (2016) and also cerebellum anterior lobe/culmen, lingual gyri, precuneus and left cuneus in the work of Wang et al. (2017). A recent study of our research group, with the same task implemented here, similarly identified overactive brain regions to fearful faces among migraineurs versus healthy controls in right middle frontal gyrus and frontal pole; and also showed increased activation to fear in association with migraine frequency in regions including right precentral and postcentral gyri (Szabó et al., 2019). All these studies made group comparisons between migraineurs and healthy controls, while we used migraine subgroups, so it is hard to compare our results with those previous ones. Nevertheless, our results overlap with the mentioned fMRI data in two ways: (1) we also found cerebral overactivation in case of a negative emotion, namely fear in both of our studies; and (2) the identified brain areas with increased activation included three regions that were connected to hypersensitivity to aversive emotional stimuli, specifically: in Study 1, left PCC (previously in Wilcox et al., 2016) and right precuneus (previously in Wilcox et al., 2016; Wang et al., 2017); while in Study 2, right precentral gyrus (previously in Szabó et al., 2019). Interestingly, both the PCC and adjacent precuneus are important parts of the default mode network (Raichle, 2015) representing cortical midline structures which have been associated with self-referential processing and self-focus (Northoff et al., 2006; Nejad et al., 2013) and in case of PCC, also the assessment of self-relevance of emotional stimuli (Vogt, 2005).

Sadness is also a negative emotion, but only fear was associated with an enhanced neural response in both of our studies. This specific role of fearful faces is not surprising, because fearful faces represent a threat stimuli and are evaluated even without awareness, gaining prioritized access to conscious visual processing (Hedger et al., 2015). Among the identified brain regions with increased activation in the Evening start group (in Study 1), superior temporal gyrus was previously shown to have a positive trend of activation in response to facial expressions with increasing intensity of fear among healthy controls (but not schizophrenic patients) (Radua et al., 2010). Higher attention to fear was also reflected by behavioral results in both of our studies: fearful faces evoked higher reaction time compared to neutral (Studies 1 and 2) and happy faces (Study 1). Slower reaction to fear is in line with previous interpretations of similar results: procession of fearful faces can lead to increased vigilance to detect the potential threat in the environment which can slow down response speed (Whalen, 1998; Davis and Whalen, 2001; Mirabella, 2018).

Interestingly, in Study 2, sad faces also associated with higher reaction time compared to neutral and happy faces. Furthermore, the accuracy rate was lower in case of sad faces in comparison with all the other conditions and higher among the Evening start group compared to the other two subgroups (Study 2). However, this slower and less accurate response to sad faces and the differences between *M*_circ_ subgroups did not correlate with alterations at a neural level.

### Pain Processing in Migraine

In a broader context, regions identified in our studies with increased activation may be also related to processing of other aversive or threatening stimuli, including pain.

The superior temporal gyrus (Study 1) is among regions that show typically different activation in response to pain among migraineurs (for a review see Schwedt et al., 2015). Other pain processing regions, many identified in previous migraine-studies, were also found in our studies. The MCC (Study 1) is a pain processing area, its increased activation among migraineurs was found in studies using painful stimuli (Stankewitz et al., 2013; Schwedt et al., 2014). The PCC is not related to direct physical pain, rather it is involved in secondary processing of psychological pain (Meerwijk et al., 2013; Wilcox et al., 2016). The pain processing network (PPN) includes the precentral (Study 2) and postcentral gyri (containing Rolandic operculum) (Study 1), and both were found to have different pain-induced activations in migraineurs compared to controls, interictally (Schwedt et al., 2015). The SMA (Study 2) is also part of the pain matrix, its pain-induced activation is thought to alert the body to move away from pain (Schwedt et al., 2014). The supramarginal gyrus (Study 1) is activated to intranasal ammonia (Stankewitz et al., 2013; Schwedt et al., 2015) and was found in many functional connectivity studies of migraine (Schwedt et al., 2015). These data belong to the numerous fMRI results suggesting an elevated pain sensitivity interictally among migraineurs as a consequence of recurrent painful attacks or migraine-associated prolonged pain (Schwedt et al., 2015). Increased activation interictally in all these pain processing regions in our study without using painful stimuli may suggest that these brain regions are more sensitive to threatening emotional stimuli, not just pain, in migraineurs with later typical circadian attack onset peak compared to the Morning start group (according to Study 1) and with earlier typical circadian attack onset peak compared to the Varying start group (according to Study 2).

### The Potential Role of Multisensory Integration in Migraine

In Study 1, increased activation was also found in right Heschl’s gyrus (or temporal transverse gyrus) containing the human primary auditory cortex (Warrier et al., 2009). Similarly to hypersensitivities to pain and other sensory stimuli, phonophobia is most prominent during attacks, but also detectable interictally with decreased intensity among many migraineurs (Schwedt, 2013). Different modes of sensory stimuli are not processed in isolation, but rather in a simultaneous way, creating an integrated perception of the environment during a process called multisensory integration which may be relevant in migraine pathophysiology (Schwedt, 2013). For example, the superior temporal gyrus is involved in auditory processing (Gernsbacher and Kaschak, 2003) and was related to olfactory processing among migraineurs, together with PCC (Demarquay et al., 2008). In our study, we found increased activation in the right precuneus that previously showed greater activation to visual stimuli in migraineurs compared to healthy controls (Griebe et al., 2014), also in the supramarginal gyrus which together with the adjacent angular gyrus form the inferior parietal lobule (also known as ventral parietal cortex) which supports higher cognitive functions where multimodal sensory (including somatosensory, proprioceptive, auditory and visual) information converge (Catani et al., 2017). Interestingly, this higher order function of the inferior parietal lobule was also detected in decoding high level features of dynamic emotional faces (Sarkheil et al., 2013; Vollstädt-Klein et al., 2019). Thus, besides auditory processing, regions involved in somatosensory, olfactory, visual and multisensory processing also showed increased activation in migraineurs with later typical circadian attack onset peak in Study 1 – suggesting an elevated level of sensory perception during the procession of fearful faces.

In Study 2, frontal lobe areas with motor functions were found to show higher activation among the Morning start migraineurs compared to the Varying start group. SMA and precentral gyrus were already discussed regarding their pain-related roles. The paracentral lobule (PCL) contains the primary motor and sensory regions for lower limbs and genitalia (Johns, 2014) and recently, its higher activation was shown during migraine episode compared to interictal state (Lei and Zhang, 2021). The PCL is part of the sensorimotor network (SMN) (which also includes precuneus), an associative cortex which have an important role in multisensory integration, too (Lei and Zhang, 2021). The observed increased PCL activation might suggest an elevated sensory processing of fearful stimuli in the Morning start group compared to the Varying start group.

In summary, in Study 1, areas with increased activation in response to fearful stimuli in the Evening start group compared to the Morning start group are involved in emotional, self-referential, pain and sensory processing. Some of the identified regions represent all or many of these functions, especially PCC and superior temporal gyrus. In Study 2, in the Morning start group, regions with similar pain and multisensory functions also showed increased activation to fearful faces compared to the Varying start group.

### Circadian Factors in Migraine Attack Onset

Appearance of emotional, pain-related and sensory stimuli during the day and their following processing may all be influenced by the circadian clock mechanism (Kim et al., 2017; Segal et al., 2018). Diurnal distribution of these and other similar factors might have an important role in migraine attack onset. For instance, it has been shown with various painful stimuli that perceived pain intensity peaks early in the morning and some studies also suggest that morning migraines are accompanied with more severe symptoms compared to migraine attacks at other times (Hsu et al., 1977; Kowanko et al., 1981; Göbel and Cordes, 1990; Gori et al., 2015; Park et al., 2018). Interestingly, a circadian variation was consistently detected for positive affective states but not for negative affect (Wood and Magnello, 1992; Murray et al., 2002; Bódizs et al., 2010) suggesting that negative affect might be more related to environmental factors (Wood and Magnello, 1992). Negative affect-related environmental effects, especially stress, are known migraine triggers and some authors suggested an environment-dependent or social nature of diurnal migraine attack onset: as we mentioned before, excessive sleep or sleep deprivation are more likely to contribute to morning migraine attacks, while work- or school-related stress to an afternoon or evening onset (Soriani et al., 2006; Alstadhaug et al., 2008; Park et al., 2018). However, in an arctic population, insomnia-related migraine attacks showed a biphasic diurnal pattern (one peak in the morning and another one in the afternoon) while not insomnia-related attacks peaked only in the afternoon (Alstadhaug et al., 2007). Regarding the circadian variation of migraine attack onset, an interaction between environment-dependent migraine triggers and the innate circadian clock mechanism is also possible (Park et al., 2018).

Recognizing the relevance of circadian variation of migraine attack onset might also contribute to migraine therapy. For instance, administration of pharmacological therapy to the typical circadian attack onset peak of the migraine patient could help prevent attacks and be a step in the direction of precision medicine. Successful implementation of this opportunity was already presented by the design of a pulsatile press coated drug delivery system containing sumatriptan succinate which was created specifically to achieve drug delivery in the early morning hours using a bedtime administration in order to prevent early morning migraine attacks (Jagdale and Pawar, 2014).

### Methodological Differences Between the Two Studies

Differences between the results from the two studies might have been originated partly from methodological differences between Study 1 and Study 2. Distinct methods were used to capture typical circadian attack onset. Answers to the self-reported question may be more subjective and deceptive than completing the headache diary. Beyond retrospective memory bias, the participants may fail to discriminate between migraines and non-migraine headaches. Furthermore, most of the studies using headache diaries even miss to elaborate on how the authors accounted for whether the reported headaches represent phenotypically migraine attacks (Poulsen et al., 2021). Furthermore, this differentiation is not obvious. For example, a previous study (Park et al., 2018) with headache diary classified migraine-type headaches based on criteria A, C, and D for migraine without aura in ICHD-3 beta, but not applied criterion B because headache duration may be significantly affected by acute migraine treatment. We decided to use a quite rigorous categorization to identify migraine headaches in the headache diary also taking account of the effect of medication (for full details, see Supplementary Appendix 1). Future studies definitely should include exact migraine attack criteria to use in headache diaries to differentiate between migraines and non-migraine headaches. The study of Park et al. (2018) also showed differences between the circadian variation of occurrence of migraine-type and non-migraine headaches.

Another important methodological difference between the two studies is the use of different MRI scanners. Recently, more and more multisite fMRI studies are conducted with different MRI scanners to enhance statistical power (Gee et al., 2015; Noble et al., 2017; Pornpattananangkul et al., 2019). However, it is known that MRI scanners from different manufacturers vary in details of construction and operation and this will be reflected in performance differences which can affect the analysis (e.g., through a main effect of scanner) causing a lower signal-to-noise ratio. Many multisite fMRI studies reported substantial site- or scanner-related effects (for details, see the work of Yu et al., 2018). This non-biological source of variation can be even more robust in case of significant site-related differences, including differences in study group ratios (Gee et al., 2015). Since we detected significant differences between the two studies in distribution of the *M*_circ_ subgroups and our study designs also differed in the methods to capture typical circadian attack onset, we decided to analyze the data from the two studies separately.

At the same time, our two samples and the *M*_circ_ subgroups showed high similarity regarding most measured descriptive variables (sex, headache frequency, sleeping problems, chronotype), only the age of the Varying start group was slightly higher in Sample 1 compared to Sample 2. Furthermore, our results survived correction for all of these factors.

## Limitations

Main limitation of our study is the low sample size limiting statistical power and generalizability of our results. Furthermore, unequal subsample sizes also might have affected our results – especially in Study 2, where only nominally significant results were found. However, we were able to show differences in neural activity between migraine subgroups, even after correcting for many relevant covariates. Our work demonstrates that besides case-control studies, investigations comparing migraine subgroups are also important because of the heterogeneous nature of migraine.

We used an implicit emotional processing fMRI task, comparing emotional (fearful, happy, and sad) facial stimuli to neutral ones which is a widely used method to control simple perceptual effects (Sabatinelli et al., 2011) and the main effect of the task processing different emotions was in line with previous meta-analysis data (Fusar-Poli et al., 2009; Lindquist et al., 2012) in both studies. The same or similar emotional processing task can be used in different ways with potential consequences on the results – recent studies with a Go/No-go facial emotional processing task showed significant differences in behavioral outcomes (including reaction time and error rate) of task-relevant versus task-irrelevant (i.e., implicit) facial emotional processing (Mirabella, 2018; Mancini et al., 2020, 2021). In future fMRI studies, it would be interesting to test the effect of the context of emotional processing in a similar way in relation to neural processing.

A cross-sectional design was used, therefore the causative effect of circadian variation of migraine attack onset on neural activity could not be investigated. In Study 1, we did not use a headache diary to measure typical circadian attack onset peak. Chronotype and sleeping problems were also captured with simple self-report measures in both studies. In Study 2, our participants filled headache diaries, however, with variation in participation time. Seasonal variation of migraine attacks also might have affected diary data. At the same time, we used exact and quite rigorous migraine attack criteria to differentiate between migraine- and non-migraine-type headaches. Another strength of our study is the proper medical diagnosis of migraine by headache specialists. Furthermore, all our subjects were thoroughly screened for chronic medical, neurological (besides migraine) and psychiatric disorders – so, we can also exclude the effect of comorbidities.

One could argue with our typical circadian attack onset peak categorization. Typically, 6-h long time slots are used, but we decided to merge the first two slots (i.e., 00:00–06:00 and 06:00–12:00) to capture the first half of the day and the last two (i.e., 12:00–18:00 and 18:00–00:00) representing the second half of the day to gain larger sample sizes. Additionally, this type of categorization is not unprecedented (Shin et al., 2015). Of course, future fMRI studies with bigger sample sizes could reveal more detailed results using four time slots.

We adjusted for the effect of age in all our analyses, but considering the association of age with both migraine and circadian rhythms (Kelman, 2006; Duffy et al., 2015; Hood and Amir, 2017), an age-stratified analysis to capture potentially age-related factors in diurnal migraine attack onset would be an interesting topic to address in future studies with bigger sample sizes and diverse age groups.

Finally, MRI scans were timed in the late afternoon/early evening hours. Investigations with a scan session in the morning/forenoon hours are needed to understand the possible effect of the timing of MRI scans on neural activity of migraine subgroups with different typical circadian attack onset peaks.

## Conclusion

According to our knowledge, this is the first investigation that tried to unfold potential biological mechanisms behind the observed phenomena of the diurnal distribution of migraine attacks. Migraineurs with very similar characteristics were grouped based on a simple circadian factor: their typical circadian attack onset peak, and this distinction associated with brain activity differences. Although, in Study 2 we could not replicate our results from Study 1, we consider our investigation as a promising first step to capture such an association since, despite the significant methodological differences between Study 1 and 2, our results from the two studies showed some important overlaps suggesting a similar mechanism: morning start migraineurs showed different brain activation patterns in both studies related specifically to fear, in regions important to emotional, pain and sensory processing-related functions. At the moment, it is highly difficult and probably too early to make conclusions about potential functional brain processes in association with circadian variation of migraine attack onset, nevertheless, our results suggest that circadian variation of migraine attack onset reflects migraine heterogeneity, and represents an important characteristic to address in future studies and prophylactic treatment of migraine.

## Data Availability Statement

The original contributions presented in the study are included in the article/Supplementary Material, further inquiries can be directed to the corresponding author/s.

## Ethics Statement

The studies involving human participants were reviewed and approved by the Scientific and Research Ethics Committee of the Medical Research Council (Hungary). The patients/participants provided their written informed consent to participate in this study.

## Author Contributions

GJ and GK conceived and designed the study. ES, NK, AG, AE, DP, TZ, MM, KG, DD, and DB were responsible for subject recruitment and data collection. DB performed the data analysis with special assistance from ES, GJ, GK, and LK. GJ, GK, GB, KG, and DD contributed to the interpretation of the data. DB wrote the first draft of the manuscript. All authors contributed to and have approved the final manuscript.

## Conflict of Interest

Preliminary data from this study were presented at the 5th Conference of the European Society for Cognitive and Affective Neuroscience, 23–26 June 2021, Online (poster presentation); and at the 33rd ECNP Congress, 12–15 September 2020, Virtual (poster presentation) and the related abstract was published in *European Neuropsychopharmacology* Volume 40, Supplement 1, November 2020, Pages S241–S242. GB is a member of the Board of Directors at Gedeon Richter and AE is an employee of Gedeon Richter Plc. Medical Division, but the company did not provide any funding or had any further role in the preparation of the article. The remaining authors declare that the research was conducted in the absence of any commercial or financial relationships that could be construed as a potential conflict of interest.

## Publisher’s Note

All claims expressed in this article are solely those of the authors and do not necessarily represent those of their affiliated organizations, or those of the publisher, the editors and the reviewers. Any product that may be evaluated in this article, or claim that may be made by its manufacturer, is not guaranteed or endorsed by the publisher.
