# Supplementary material for: Circadian Variation of Migraine Attack Onset Affects fMRI Brain Response to Fearful Faces
Source: Front Hum Neurosci. 2022 Mar 9;16:842426. doi: 10.3389/fnhum.2022.842426 (PMC8959375; doi:10.3389/fnhum.2022.842426)
Supplement: Supplementary file 1 [file Data_Sheet_1.docx]

**Supplementary Material**

Circadian variation of migraine attack onset affects fMRI brain response to fearful faces

**Daniel Baksa, Edina Szabo, Natalia Kocsel, Attila Galambos, Andrea Edit Edes, Dorottya Pap, Terezia Zsombok, Mate Magyar, Kinga Gecse, Dora Dobos, Lajos Rudolf Kozak, Gyorgy Bagdy, Gyongyi Kokonyei, Gabriella Juhasz**

**Supplementary Appendix 1**. Headache diary, migraine attack and typical circadian attack onset peak criteria in Study 2

**Headache diary:**

| Month: | 1 | 2 | 3 | 4 | 5 | 6 | 7 | 8 | 9 | 10 | 11 | 12 | 13 | 14 | 15 | 16 | 17 | 18 | 19 | 20 | 21 | 22 | 23 | 24 | 25 | 26 | 27 | 28 | 29 | 30 | 31 |
| --- | --- | --- | --- | --- | --- | --- | --- | --- | --- | --- | --- | --- | --- | --- | --- | --- | --- | --- | --- | --- | --- | --- | --- | --- | --- | --- | --- | --- | --- | --- | --- |
| Headache – at what time did it start? |  |  |  |  |  |  |  |  |  |  |  |  |  |  |  |  |  |  |  |  |  |  |  |  |  |  |  |  |  |  |  |
| Location of headache  (Right/left/both) |  |  |  |  |  |  |  |  |  |  |  |  |  |  |  |  |  |  |  |  |  |  |  |  |  |  |  |  |  |  |  |
| Pain intensity * |  |  |  |  |  |  |  |  |  |  |  |  |  |  |  |  |  |  |  |  |  |  |  |  |  |  |  |  |  |  |  |
| Effect of pain ** |  |  |  |  |  |  |  |  |  |  |  |  |  |  |  |  |  |  |  |  |  |  |  |  |  |  |  |  |  |  |  |
| Pain quality *** |  |  |  |  |  |  |  |  |  |  |  |  |  |  |  |  |  |  |  |  |  |  |  |  |  |  |  |  |  |  |  |
| Nausea or vomiting **** |  |  |  |  |  |  |  |  |  |  |  |  |  |  |  |  |  |  |  |  |  |  |  |  |  |  |  |  |  |  |  |
| Photophobia **** |  |  |  |  |  |  |  |  |  |  |  |  |  |  |  |  |  |  |  |  |  |  |  |  |  |  |  |  |  |  |  |
| Phonophobia **** |  |  |  |  |  |  |  |  |  |  |  |  |  |  |  |  |  |  |  |  |  |  |  |  |  |  |  |  |  |  |  |
| Osmophobia **** |  |  |  |  |  |  |  |  |  |  |  |  |  |  |  |  |  |  |  |  |  |  |  |  |  |  |  |  |  |  |  |
| Discomfort by neutral stimuli **** |  |  |  |  |  |  |  |  |  |  |  |  |  |  |  |  |  |  |  |  |  |  |  |  |  |  |  |  |  |  |  |
| Aggravation by physical activity **** |  |  |  |  |  |  |  |  |  |  |  |  |  |  |  |  |  |  |  |  |  |  |  |  |  |  |  |  |  |  |  |
| Medication (yes/no) |  |  |  |  |  |  |  |  |  |  |  |  |  |  |  |  |  |  |  |  |  |  |  |  |  |  |  |  |  |  |  |
| Headache duration in hours |  |  |  |  |  |  |  |  |  |  |  |  |  |  |  |  |  |  |  |  |  |  |  |  |  |  |  |  |  |  |  |

*at its worst point, on a 0-10 scale

**1=mild (not affecting work) 2=moderate (affecting work) 3=strong (inhibiting work)

***Pr=pressing/squeezing Po=pounding Th=throbbing/pulsating S=stabbing Ti=tightening O=other

****+ sign if experienced besides headache, - sign if not

**Migraine attack criteria**:

at least 4 out of 6 symptom categories:

1. Duration: at least 4 hours
2. Location of headache: unilateral
3. Headache quality: any of pounding, throbbing/pulsating, stabbing
4. Headache intensity: at least 5 points on Pain intensity and/or at least moderate Effect of pain
5. Aggravation by phisical activity
6. Accompanying symptoms: any of nausea or vomiting, photophobia, phonophobia, osmophobia, discomfort by neutral stimuli

In case of medication use: at least 3 out of the 6 symptom categories.

**Typical circadian attack onset peak criteria:**

At least two migraine attacks in the headache diary (based on the migraine attack criteria described above).

Based on the answer to „Headache – at what time did it start?” in the headache diary:

- Morning start (representing the first half of the day): 00:00-11:59
- Evening start (representing the second half of the day): 12:00-23:59
- In a few cases, three participants did not provide an exact starting time but stated: “after waking up in the morning”, “morning”, “dawn” (these answers were categorized as Morning start) or “evening” (categorized as Evening start).

At least 60% of migraine attacks in the same category:

- Morning start group: 60% or more of attacks in the Morning start category.
- Evening start group: 60% or more of attacks in the Evening start category.
- Varying start group: below 60% of attacks in any of the Morning and Evening categories.

**Supplementary Table 1.** Behavioral data of Study 1 sample and statistical results of the comparison between M_circ_ subgroups and reactions to different emotions

| **Accuracy** (mean %, SD) | **Total** | **Morning start (M)** | **Evening start (E)** | **Varying start (V)** | **Group comparison:** *M vs E vs V* |
| --- | --- | --- | --- | --- | --- |
| *All conditions* | 98.76 (2.66) | 99.4 (1.4) | 98.14 (4.12) | 98.8 (1.94) | H=1.098, p=0.577 |
| *Fear* | 98.46 (3.62) | 99.3 (1.98) | 96.9 (5.65) | 99.07 (2.18) | H=1.495, p=0.473 |
| *Happy* | 98.46 (3.29) | 97.9 (2.9) | 98.77 (3.7) | 98.61 (3.45) | H=1.363, p=0.506 |
| *Sad* | 97.12 (5.69) | 98.61 (3.92) | 96.28 (7.38) | 96.75 (5.54) | H=1.049, p=0.592 |
| *Neutral* | 99.18 (2.05) | 99.65 (0.65) | 98.77 (3.21) | 99.18 (1.63) | H=0.033, p=0.983 |
| **Comparison** **of reactions to emotions**:  *Fear vs Happy vs Sad vs Neutral* | χ^2^=4.702, p=0.195 | χ^2^=2.111, p=0.55 | χ^2^=4.875, p=0.181 | χ^2^=3.867, p=0.276 |  |
| **Reaction time** (in msec; mean, SD) | **Total** | **Morning start (M)** | **Evening start (E)** | **Varying start (V)** | **Group comparison:** *M vs E vs V* |
| *All conditions* | 94418.53 (18678.73) | 98817.62 (16993.42) | 97537.56 (14110.48) | 89552.07 (22273.73) | H=2.087, p=0.352 |
| *Fear* | 790.48 (160.8) | 813.28 (121.42) | 846.93 (136.34) | 737.37 (188.94) | H=3.147, p=0.207 |
| *Happy* | 751.7 (159.7) | 801.6 (180.94) | 773.64 (112.21) | 705.8 (172.72) | H=1.752, p=0.416 |
| *Sad* | 757.48 (162.44) | 836.86 (177.36) | 751.12 (124.24) | 713.04 (169.57) | H=1.356, p=0.508 |
| *Neutral* | 736.45 (147.33) | 759.53 (123.15) | 761.76 (112.54) | 704.72 (182.32) | H=1.856, p=0.395 |
| **Comparison** **of reactions to emotions**:  *Fear vs Happy vs Sad vs Neutral* | χ^2^=14.36, p=0.002*  (fear > happy; Z=-2.602, p=0.009)*  (fear > neutral; Z=-3.671, p<0.001)* | χ^2^=8.85, p=0.031*  (fear > neutral; Z=-2.24, p=0.025)*  (sad > neutral; Z=-2.521, p=0.012)* | χ^2^=9.4, p=0.024* (fear > sad; Z=-2.31, p=0.021)*  (fear > neutral; Z=-2.666, p=0.008)* | χ^2^=2,631, p=0.452 |  |

*Note.* H: Kruskal-Wallis test statistic; SD: standard deviation; χ^2^: chi-square test statistic of Friedman test; Z: Wilcoxon signed-rank test statistic; *: significant effect; M_circ_ subgroups: M: Morning start; E: Evening start; V: Varying start; msec: millisecond. For each participant, accuracy rate was computed as the ratio between the numbers of correct answers (i.e. detecting the correct sex of faces) and overall answers. Reaction times were detected in milliseconds – for all conditions, the sum of all reaction times was used, while in case of each emotion (fear, happy, sad, neutral), the summed reaction time value was divided by the number of trials to make them comparable with each other. Outliers were detected and excluded based on the following criteria: over mean +/- three SDs. One participant from the Varying start group was left out from all analyses because of 0% accuracy rate. One further participant (Varying star group) was also left out from accuracy analyses because of outlying accuracy values. Exclusions resulted in sample sizes of n=29 in case of accuracy analyses, and n=30 in reaction time analyses.

**Supplementary Table 2.** Behavioral data of Study 2 sample and statistical results of the comparison between M_circ_ subgroups and reactions to different emotions

| **Accuracy** (mean %, SD) | **Total** | **Morning start (M)** | **Evening start (E)** | **Varying start (V)** | **Group comparison:** *M vs E vs V* |
| --- | --- | --- | --- | --- | --- |
| *All conditions* | 99.5 (0.77) | 99.33 (0.75) | 99.78 (0.36) | 98.9 (1.28) | H=5.421, p=0.066 |
| *Fear* | 99.75 (1.17) | 99.53 (1.62) | 99.78 (1.12) | 100 (0) | H=0.793, p=0.673 |
| *Happy* | 99.63 (1.41) | 99.53 (1.62) | 99.78 (1.12) | 99.3 (1.98) | H=0.76, p=0.684 |
| *Sad* | 98.76 (2.35) | 97.67 (2.88) | 99.78 (1.12) | 97.2 (2.99) | H=10.755, p=0.005* (E > M; U=93.5, p=0.004)*  (E > V; U=54; p=0.002)* |
| *Neutral* | 99.63 (0.81) | 99.53 (0.91) | 99.88 (0.39) | 98.95 (1.24) | H=7.403, p=0.025*  (E > V; U=56, p=0.006)* |
| **Comparison** **of reactions to emotions**:  *Fear vs Happy vs Sad vs Neutral* | χ^2^=10.765, p=0.013* (fear > sad; Z=-2.309, p=0.021)*  (happy > sad; Z=-2.111, p=0.035)*  (neutral > sad; Z=-2.691, p=0.007)* | χ^2^=5.491, p=0.139 | χ^2^=0.6, p=0.896 | χ^2^=9.25, p=0.026*  (fear > sad; Z=-2, p=0.046)* |  |
| **Reaction time** (in msec; mean, SD) | **Total** | **Morning start (M)** | **Evening start (E)** | **Varying start (V)** | **Group comparison:** *M vs E vs V* |
| *All conditions* | 102674.09 (14106.74) | 99973.5 (11976.55) | 102107.72 (14051.59) | 107848.11 (16963.12) | H=0.521, p=0.771 |
| *Fear* | 828.9 (134.38) | 813.13 (99.99) | 820.81 (127.82) | 872.43 (190.03) | H=0.301, p=0.86 |
| *Happy* | 806.83 (109.39) | 794.67 (105.58) | 808.94 (124.07) | 817.19 (73.82) | H=0.318, p=0.853 |
| *Sad* | 845.36 (128.86) | 815.49 (120.77) | 848.2 (140.16) | 877.31 (109.07) | H=1.002, p=0.606 |
| *Neutral* | 805.75 (113.89) | 782.7 (89.93) | 798.67 (106.5) | 856.16 (154.94) | H=0.521, p=0.771 |
| **Comparison** **of reactions to emotions**:  *Fear vs Happy vs Sad vs Neutral* | χ^2^=13.174, p=0.004*  (fear > neutral; Z=-2.322, p=0.02)*  (sad > happy; Z=-3.13, p=0.002)*  (sad > neutral; Z=-3.676, p<0.001)* | χ^2^=4.9, p=0.179 | χ^2^=4.73, p=0.193 | χ^2^=8.6, p=0.035*  (sad > happy; Z=-2.666, p=0.008)* |  |

*Note.* H: Kruskal-Wallis test statistic; SD: standard deviation; U: Mann-Whitney test statistic; χ^2^: chi-square test statistic of Friedman test; Z: Wilcoxon signed-rank test statistic; *: significant effect; M_circ_ subgroups: M: Morning start; E: Evening start; V: Varying start; msec: millisecond. For each participant, accuracy rate was computed as the ratio between the numbers of correct answers (i.e. detecting the correct sex of faces) and overall answers. Reaction times were detected in milliseconds – for all conditions, the sum of all reaction times was used, while in case of each emotion (fear, happy, sad, neutral), the summed reaction time value was divided by the number of trials to make them comparable with each other. Outliers were detected and excluded based on the following criteria: over mean +/- three SDs. One participant from each M_circ_ subgroups was left out because of low accuracy rates. Two participants (one from Evening start group, another one from Morning start group) were left out because of outlying reaction time values. Exclusions resulted in sample sizes of n=45 in case of accuracy analyses, and n=46 in reaction time analyses.

**Supplementary Table 3.** Comparison of self-reported data between total samples and M_circ_ subgroups of Study 1 and Study 2

|  | **Total** | | | **Morning start** | | | **Evening start** | | | **Varying start** | | |
| --- | --- | --- | --- | --- | --- | --- | --- | --- | --- | --- | --- | --- |
|  | *Study 1* | *Study 2* | *Test statistic* | *Study 1* | *Study 2* | *Test statistic* | *Study 1* | *Study 2* | *Test statistic* | *Study 1* | *Study 2* | *Test statistic* |
| **Participant number** (n) | 31 | 48 | - | 8 | 13 | χ^2^=7.21, p=0.027* (Study 1 < Study 2) | 9 | 26 | χ^2^=7.21, p=0.027* (Study 1 < Study 2) | 14 | 9 | χ^2^=7.21, p=0.027* (Study 1 > Study 2) |
| **Sex** (n) |  |  |  |  |  |  |  |  |  |  |  |  |
| *Female* | 24 | 43 | Fisher’s exact p=0.2 | 7 | 11 | Fisher’s exact p=1 | 6 | 24 | Fisher’s exact p=0.095 | 11 | 8 | Fisher’s exact p=1 |
| *Male* | 7 | 5 |  | 1 | 2 |  | 3 | 2 |  | 3 | 1 |  |
| **Age** (mean, SD) | 26.97 (4.83) | 27.02 (6.29) | U=701.5,  p=0.669 | 26.12 (4.32) | 31.23 (7.81) | U=32,  p=0.146 | 23.67 (2) | 25.62 (5.2) | U=99.5,  p=0.506 | 29.57 (5.1) | 25 (4.09) | U=30,  p=0.037* |
| **Self-reported attack frequency per month** (mean, SD) | 3.34 (3.15) | 3.06 (2.68) | U=718,  p=0.791 | 2.31 (1.13) | 2.77 (2.1) | U=51,  p=0.941 | 4.55 (4.44) | 3.02 (2.73) | U=102.5, p=0.577 | 3.14 (2.88) | 3.62 (3.46) | U=62,  p=0.949 |
| **Chronotype** (n) |  |  |  |  |  |  |  |  |  |  |  |  |
| *Definitely / rather morning* | 13 | 18 | Fisher’s exact p=0.917 | 4 | 6 | Fisher’s exact p=0.486 | 2 | 7 | Fisher’s exact p=1 | 7 | 5 | Fisher’s exact p=1 |
| *Definitely / rather evening* | 17 | 28 |  | 3 | 7 |  | 7 | 17 |  | 7 | 4 |  |
| *Do not know* | 1 | 2 |  | 1 | 0 |  | 0 | 2 |  | 0 | 0 |  |
| **Sleeping problems** (n) |  |  |  |  |  |  |  |  |  |  |  |  |
| *never/rarely* | 14 | 26 | χ^2^=1.127,  p=0.569 | 4 | 6 | Fisher’s exact p=1 | 4 | 17 | Fisher’s exact p=0.479 | 6 | 3 | Fisher’s exact p=1 |
| *sometimes* | 14 | 16 |  | 4 | 6 |  | 4 | 6 |  | 6 | 4 |  |
| *often/usually* | 3 | 6 |  | 0 | 1 |  | 1 | 3 |  | 2 | 2 |  |

*Note.* SD: standard deviation; U: Mann-Whitney test statistic; χ^2^: chi-square test statistic; *: significant.

**Supplementary Table 4**. Main effect of task in all participants in Study 1

| **Contrast** | **Activation** | **Cluster size** | **Cluster *p* (FWE)** | **Region** | **Coordinates (MNI)** | | | **Peak t-value** |
| --- | --- | --- | --- | --- | --- | --- | --- | --- |
|  |  |  |  |  | **x** | **y** | **z** |  |
| ***Fear-neutral*** | ***Increased*** |  |  |  |  |  |  |  |
|  |  | 4247 | <0.001 | R inferior temporal gyrus | 45 | -46 | -16 | 11.37 |
|  |  |  |  | L fusiform gyrus | -36 | -55 | -13 | 11.35 |
|  |  |  |  | R inferior temporal gyrus | 45 | -55 | -13 | 10.97 |
|  |  |  |  | L middle occipital gyrus | -24 | -94 | 8 | 10.91 |
|  |  |  |  | L fusiform gyrus | -33 | -73 | -13 | 10.67 |
|  |  |  |  | L middle occipital gyrus | -27 | -85 | 8 | 10.62 |
|  |  |  |  | R inferior occipital gyrus | 42 | -70 | -16 | 10.28 |
|  |  |  |  | L fusiform gyrus | -30 | -76 | -19 | 10.23 |
|  |  |  |  | L fusiform gyrus | -39 | -67 | -16 | 10.15 |
|  |  |  |  | L inferior occipital gyrus | -39 | -79 | -10 | 10.13 |
|  |  |  |  | R middle occipital gyrus | 39 | -85 | -1 | 9.92 |
|  |  |  |  | R occipital fusiform gyrus | 36 | -61 | -7 | 9.85 |
|  |  |  |  | R inferior occipital gyrus | 42 | -82 | -4 | 9.37 |
|  |  |  |  | L lingual gyrus | -15 | -88 | -10 | 9.37 |
|  |  |  |  | R inferior occipital gyrus | 36 | -70 | -7 | 9.35 |
|  |  |  |  | R middle occipital gyrus | 33 | -88 | 5 | 8.94 |
|  |  | 107 | 0.004 | L inferior frontal gyrus, orbital part | -48 | 41 | -4 | 6.23 |
|  |  |  |  | L inferior frontal gyrus, orbital part | -42 | 26 | -16 | 4.99 |
|  |  |  |  | L inferior frontal gyrus, orbital part | -45 | 29 | -13 | 4.17 |
|  |  |  |  | L inferior frontal gyrus, orbital part | -51 | 29 | -10 | 4.05 |
|  |  | 176 | <0.001 | R amygdala | 21 | -7 | -13 | 6.18 |
|  |  |  |  | R amygdala | 30 | -4 | -19 | 5.26 |
|  |  |  |  | R putamen | 33 | -10 | -10 | 4.16 |
|  |  |  |  | R hippocampus | 30 | -22 | -4 | 3.86 |
|  |  | 528 | <0.001 | R inferior frontal gyrus, opercular part | 48 | 11 | 35 | 5.94 |
|  |  |  |  | R inferior frontal gyrus, triangular part | 45 | 26 | 23 | 5.91 |
|  |  |  |  | R inferior frontal gyrus, opercular part | 45 | 11 | 29 | 5.83 |
|  |  |  |  | R inferior frontal gyrus, triangular part | 51 | 32 | 29 | 5.63 |
|  |  |  |  | R inferior frontal gyrus, triangular part | 57 | 23 | 26 | 5.56 |
|  |  | 96 | 0.006 | L amygdala | -21 | -4 | -16 | 5.86 |
|  |  | 194 | <0.001 | L inferior frontal gyrus, triangular part | -51 | 26 | 26 | 5.31 |
|  |  |  |  | L inferior frontal gyrus, triangular part | -48 | 20 | 29 | 5.27 |
|  |  |  |  | L inferior frontal gyrus, triangular part | -54 | 29 | 20 | 5.25 |
|  |  |  |  | L inferior frontal gyrus, triangular part | -57 | 26 | 14 | 4.58 |
|  |  |  |  | L middle frontal gyrus | -36 | 5 | 35 | 4.27 |
|  |  |  |  | L middle frontal gyrus | -36 | 11 | 35 | 4.2 |
|  |  | 90 | 0.009 | L middle cingulate gyrus | -3 | 38 | 50 | 5.05 |
|  |  |  |  | R supplementary motor area | 9 | 23 | 53 | 4.94 |
|  |  |  |  | L supplementary motor area | -6 | 23 | 50 | 4.81 |
| ***Fear-neutral*** | ***Decreased*** |  |  |  |  |  |  |  |
|  |  | 99 | 0.006 | R precuneus | 21 | -46 | 8 | 4.73 |
|  |  | 77 | 0.017 | L precuneus | -21 | -49 | 5 | 4.77 |
|  |  | 130 | 0.001 | R middle cingulate gyrus | 3 | -43 | 41 | 4.7 |
|  |  |  |  | R middle cingulate gyrus | 6 | -34 | 41 | 4.55 |
| ***Happy-neutral*** | ***Increased*** |  |  |  |  |  |  |  |
|  |  | 1249 | <0.001 | L middle occipital gyrus | -27 | -88 | 11 | 8.4 |
|  |  |  |  | L inferior occiptal gyrus | -39 | -79 | -7 | 6.83 |
|  |  |  |  | L occipital fusiform gyrus | -30 | -70 | -4 | 6.52 |
|  |  |  |  | L middle occipital gyrus | -33 | -76 | -1 | 6.52 |
|  |  |  |  | L fusiform gyrus | -33 | -61 | -7 | 6.39 |
|  |  |  |  | L inferior occiptal gyrus | -33 | -73 | -7 | 6.35 |
|  |  |  |  | L middle occipital gyrus | -36 | -79 | 5 | 6.34 |
|  |  |  |  | L inferior occiptal gyrus | -39 | -64 | -7 | 6.24 |
|  |  |  |  | L inferior occiptal gyrus | -39 | -70 | -10 | 6.23 |
|  |  |  |  | L middle occipital gyrus | -15 | -94 | -1 | 6.17 |
|  |  |  |  | L fusiform gyrus | -42 | -58 | -16 | 6.08 |
|  |  |  |  | L fusiform gyrus | -27 | -61 | -10 | 5.73 |
|  |  |  |  | L inferior temporal gyrus | -45 | -49 | -19 | 5.52 |
|  |  |  |  | L inferior occiptal gyrus | -27 | -88 | -7 | 5.32 |
|  |  |  |  | L inferior occiptal gyrus | -33 | -88 | -7 | 5.26 |
|  |  |  |  | L middle occipital gyrus | -27 | -82 | 35 | 4.87 |
|  |  | 1776 | <0.001 | R middle occipital gyrus | 24 | -88 | 8 | 7.89 |
|  |  |  |  | R fusiform gyrus | 39 | -49 | -16 | 7.02 |
|  |  |  |  | R inferior occipital gyrus | 36 | -64 | -10 | 6.95 |
|  |  |  |  | R fusiform gyrus | 39 | -37 | -22 | 6.91 |
|  |  |  |  | R inferior temporal gyrus | 48 | -67 | -7 | 5.92 |
|  |  |  |  | R cerebellum exterior | 24 | -37 | -25 | 5.79 |
|  |  |  |  | R lingual gyrus | 6 | -73 | -1 | 5.14 |
|  |  |  |  | R calcarine cortex | 18 | -76 | 11 | 4.88 |
|  |  |  |  | R middle occipital gyrus | 30 | -70 | 29 | 4.47 |
|  |  |  |  | R superior occipital gyrus | 30 | -76 | 41 | 4.11 |
|  |  |  |  | R middle temporal gyrus | 42 | -70 | 17 | 4.08 |
|  |  |  |  | brain stem | 9 | -34 | -28 | 4.04 |
|  |  |  |  | R middle temporal gyrus | 54 | -58 | 11 | 3.91 |
|  |  |  |  | R middle temporal gyrus | 51 | -73 | 8 | 3.74 |
|  |  |  |  | brain stem | -6 | -37 | -31 | 3.72 |
|  |  |  |  | brain stem | 3 | -34 | -31 | 3.72 |
|  |  | 175 | <0.001 | R amygdala | 24 | -1 | -13 | 5.88 |
|  |  |  |  | R superior temporal pole | 30 | 5 | -22 | 5.03 |
|  |  |  |  | R hippocampus | 27 | -16 | -10 | 4.8 |
|  |  | 79 | 0.014 | L amygdala | -24 | -4 | -22 | 5.65 |
|  |  |  |  | L amygdala | -27 | -1 | -16 | 4.72 |
|  |  |  |  | L putamen | -15 | 8 | -13 | 3.78 |
|  |  | 161 | <0.001 | R inferior frontal gyrus, triangular part | 42 | 32 | 11 | 4.97 |
|  |  |  |  | R inferior frontal gyrus, triangular part | 42 | 26 | 20 | 4.92 |
|  |  |  |  | R inferior frontal gyrus, triangular part | 48 | 32 | 14 | 4.84 |
|  |  |  |  | R inferior frontal gyrus, triangular part | 48 | 26 | 20 | 4.58 |
|  |  |  |  | R inferior frontal gyrus, opercular part | 42 | 5 | 23 | 4.48 |
|  |  |  |  | R inferior frontal gyrus, opercular part | 39 | 8 | 29 | 4.47 |
|  |  |  |  | R middle frontal gyrus | 39 | 8 | 35 | 4.47 |
|  |  |  |  | R inferior frontal gyrus, triangular part | 54 | 29 | 20 | 4.19 |
|  |  |  |  | R inferior frontal gyrus, triangular part | 57 | 23 | 23 | 4.07 |
| ***Sad-neutral*** | ***Increased*** |  |  |  |  |  |  |  |
|  |  | 1682 | <0.001 | L fusiform gyrus | -27 | -70 | -7 | 5.74 |
|  |  |  |  | R middle occipital gyrus | 36 | -82 | 2 | 5.48 |
|  |  |  |  | L middle occipital gyrus | -30 | -85 | 17 | 5.45 |
|  |  |  |  | L lingual gyrus | -18 | -82 | -13 | 5.37 |
|  |  |  |  | L lingual gyrus | -27 | -61 | -7 | 5.29 |
|  |  |  |  | L superior occipital gyrus | -15 | -94 | 17 | 5.18 |
|  |  |  |  | R inferior temporal gyrus | 45 | -52 | -13 | 5.12 |
|  |  |  |  | L fusiform gyrus | -45 | -52 | -16 | 5.05 |
|  |  |  |  | L inferior temporal gyrus | -42 | -49 | -7 | 4.99 |
|  |  |  |  | L middle occipital gyrus | -39 | -85 | 5 | 4.92 |
|  |  |  |  | R superior occipital gyrus | 21 | -91 | 20 | 4.83 |
|  |  |  |  | R fusiform gyrus | 42 | -37 | -19 | 4.8 |
|  |  |  |  | R fusiform gyrus | 33 | -61 | -10 | 4.64 |
|  |  |  |  | R lingual gyrus | 18 | -79 | -13 | 4.59 |
|  |  |  |  | R inferior occipital gyrus | 39 | -67 | -10 | 4.52 |
|  |  |  |  | R lingual gyrus | 9 | -85 | -10 | 4.5 |
|  |  | 70 | 0.035 | R inferior frontal gyrus, triangular part | 51 | 32 | 14 | 4.69 |
|  |  |  |  | R inferior frontal gyrus, opercular part | 39 | 17 | 17 | 4.32 |
|  |  |  |  | R middle frontal gyrus | 39 | 23 | 17 | 4.04 |
|  |  |  |  | R inferior frontal gyrus, opercular part | 39 | 8 | 26 | 3.82 |
| ***Sad-neutral*** | ***Decreased*** |  |  |  |  |  |  |  |
|  |  | 74 | 0.029 | L superior temporal gyrus | -42 | -40 | 17 | 4.88 |
|  |  |  |  | L superior temporal gyrus | -63 | -31 | 14 | 4.47 |

*Note.* Cluster *p* (FWE): cluster level family-wise error corrected p-value; L: Left hemisphere; R: right hemisphere; MNI: coordinates in Montreal Neurological Institute (MNI) space, Peak t-value: peak test-statistic of the one-sample t-test.

**Supplementary Table 5**. Main effect of task in all participants in Study 2

| **Contrast** | **Activation** | **Cluster size** | **Cluster *p* (FWE)** | **Region** | **Coordinates (MNI)** | | | **Peak t-value** |
| --- | --- | --- | --- | --- | --- | --- | --- | --- |
|  |  |  |  |  | **x** | **y** | **z** |  |
| ***Fear-neutral*** | ***Increased*** |  |  |  |  |  |  |  |
|  |  | 3340 | <0.001 | R lingual gyrus | 24 | -79 | -7 | 10.70 |
|  |  |  |  | R middle occipital gyrus | 30 | -91 | 8 | 10.49 |
|  |  |  |  | R inferior occipital gyrus | 33 | -76 | -10 | 9.06 |
|  |  |  |  | R middle occipital gyrus | 39 | -85 | -1 | 8.75 |
|  |  |  |  | L inferior temporal gyrus | -39 | -46 | -16 | 8.67 |
|  |  |  |  | R inferior occipital gyrus | 36 | -85 | -7 | 8.47 |
|  |  |  |  | L middle occipital gyrus | -27 | -91 | 8 | 8.37 |
|  |  |  |  | L fusiform gyrus | -21 | -85 | -7 | 8.37 |
|  |  |  |  | L inferior occipital gyrus | -21 | -88 | -1 | 8.23 |
|  |  |  |  | L inferior occipital gyrus | -33 | -76 | -10 | 8.14 |
|  |  |  |  | R lingual gyrus | 3 | -85 | -7 | 7.94 |
|  |  |  |  | L inferior occipital gyrus | -33 | -85 | -4 | 7.85 |
|  |  |  |  | R fusiform gyrus | 30 | -61 | -10 | 7.69 |
|  |  |  |  | R fusiform gyrus | 39 | -49 | -16 | 7.09 |
|  |  |  |  | L inferior temporal gyrus | -42 | -58 | -10 | 6.6 |
|  |  |  |  | R lingual gyrus | 12 | -91 | -4 | 6.44 |
|  |  | 587 | <0.001 | R inferior frontal gyrus, triangular part | 54 | 38 | 8 | 6.13 |
|  |  |  |  | R inferior frontal gyrus, triangular part | 42 | 17 | 23 | 5.65 |
|  |  |  |  | R inferior frontal gyrus, opercular part | 42 | 23 | 20 | 5.64 |
|  |  |  |  | R inferior frontal gyrus, opercular part | 36 | 8 | 23 | 5.57 |
|  |  |  |  | R precentral gyrus | 39 | -1 | 38 | 5.25 |
|  |  |  |  | R precentral gyrus | 45 | 2 | 32 | 4.88 |
|  |  |  |  | R middle frontal gyrus | 54 | 35 | 20 | 4.46 |
|  |  |  |  | R inferior frontal gyrus, triangular part | 39 | 32 | 2 | 4.35 |
|  |  |  |  | R inferior frontal gyrus, triangular part | 54 | 29 | 29 | 4.02 |
|  |  | 149 | 0.001 | L inferior frontal gyrus, opercular part | -33 | 5 | 29 | 5.82 |
|  |  |  |  | L frontal operculum | -36 | 20 | 17 | 3.81 |
|  |  |  |  | L inferior frontal gyrus, opercular part | -51 | 11 | 20 | 3.75 |
|  |  |  |  | L inferior frontal gyrus, triangular part | -45 | 14 | 23 | 3.73 |
|  |  |  |  | L inferior frontal gyrus, opercular part | -57 | 14 | 11 | 3.69 |
|  |  |  |  | L inferior frontal gyrus, triangular part | -42 | 17 | 20 | 3.66 |
| ***Fear-neutral*** | ***Decreased*** |  |  |  |  |  |  |  |
|  |  | 281 | <0.001 | R anterior cingulate gyrus | 9 | 35 | 11 | 5.01 |
|  |  |  |  | L anterior cingulate gyrus | -15 | 50 | 2 | 4.71 |
|  |  |  |  | L anterior cingulate gyrus | 0 | 32 | 23 | 4.61 |
|  |  |  |  | L anterior cingulate gyrus | -15 | 41 | -1 | 4.55 |
|  |  |  |  | R anterior cingulate gyrus | 6 | 32 | -4 | 4.17 |
|  |  |  |  | R medial frontal gyrus, orbital part | 6 | 41 | -7 | 4.13 |
|  |  |  |  | R anterior cingulate gyrus | 9 | 35 | -1 | 3.95 |
|  |  |  |  | R anterior cingulate gyrus | 21 | 41 | 11 | 3.67 |
|  |  |  |  | L anterior cingulate gyrus | -3 | 41 | 5 | 3.66 |
|  |  |  |  | R anterior cingulate gyrus | 6 | 44 | 17 | 3.59 |
| ***Happy-neutral*** | ***Increased*** |  |  |  |  |  |  |  |
|  |  | 1358 | <0.001 | L middle occipital gyrus | -21 | -94 | 11 | 11.59 |
|  |  |  |  | L inferior occipital gyrus | -39 | -76 | -13 | 6.96 |
|  |  |  |  | L fusiform gyrus | -36 | -55 | -16 | 6.51 |
|  |  |  |  | L inferior occipital gyrus | -39 | -85 | 2 | 6.07 |
|  |  |  |  | L fusiform gyrus | -24 | -82 | -13 | 5.49 |
|  |  |  |  | L lingual gyrus | -27 | -64 | -10 | 5.47 |
|  |  |  |  | L fusiform gyrus | -30 | -67 | -13 | 5.3 |
|  |  |  |  | L lingual gyrus | -9 | -88 | -10 | 4.13 |
|  |  | 1741 | <0.001 | R middle occipital gyrus | 30 | -85 | 8 | 6.93 |
|  |  |  |  | R superior occipital gyrus | 18 | -97 | 17 | 6.7 |
|  |  |  |  | R superior occipital gyrus | 21 | -94 | 14 | 6.69 |
|  |  |  |  | R fusiform gyrus | 36 | -52 | -13 | 6.62 |
|  |  |  |  | R fusiform gyrus | 45 | -61 | -19 | 6.39 |
|  |  |  |  | R fusiform gyrus | 42 | -52 | -19 | 6.28 |
|  |  |  |  | R inferior occipital gyrus | 42 | -76 | -7 | 5.99 |
|  |  |  |  | R fusiform gyrus | 33 | -76 | -13 | 5.99 |
|  |  |  |  | R inferior temporal gyrus | 45 | -73 | -10 | 5.9 |
|  |  |  |  | R inferior occipital gyrus | 45 | -82 | 2 | 5.51 |
|  |  |  |  | R lingual gyrus | 24 | -61 | -7 | 4.95 |
|  |  |  |  | R middle temporal gyrus | 51 | -58 | 8 | 4.86 |
|  |  |  |  | R superior parietal lobule | 36 | -61 | 56 | 4.6 |
|  |  |  |  | R angular gyrus | 27 | -55 | 44 | 4.29 |
|  |  |  |  | R fusiform gyrus | 42 | -34 | -16 | 4.1 |
|  |  |  |  | R middle temporal gyrus | 60 | -58 | 5 | 3.98 |
|  |  | 96 | 0.013 | L Cerebellum exterior | -9 | -76 | -40 |  |
|  |  |  |  | L Cerebellum exterior | -18 | -70 | -49 |  |
|  |  |  |  | L Cerebellum exterior | -24 | -67 | -49 |  |
| ***Sad-neutral*** | ***Increased*** |  |  |  |  |  |  |  |
|  |  | 7097 | <0.001 | R fusiform gyrus | 24 | -76 | -10 | 8.65 |
|  |  |  |  | R middle occipital gyrus | 45 | -76 | -1 | 7.98 |
|  |  |  |  | R fusiform gyrus | 39 | -34 | -19 | 7.91 |
|  |  |  |  | L fusiform gyrus | -24 | -70 | -7 | 7.86 |
|  |  |  |  | L fusiform gyrus | -24 | -79 | -10 | 7.81 |
|  |  |  |  | L lingual gyrus | -21 | -82 | -7 | 7.8 |
|  |  |  |  | L superior occipital gyrus | -24 | -94 | 23 | 7.78 |
|  |  |  |  | L middle occipital gyrus | -36 | -88 | 11 | 7.77 |
|  |  |  |  | L middle occipital gyrus | -33 | -88 | 17 | 7.77 |
|  |  |  |  | R calcarine cortex | 12 | -94 | 11 | 7.74 |
|  |  |  |  | L middle occipital gyrus | -30 | -85 | 8 | 7.67 |
|  |  |  |  | L fusiform gyrus | -21 | -82 | -13 | 7.66 |
|  |  |  |  | L middle occipital gyrus | -39 | -85 | 2 | 7.65 |
|  |  |  |  | L fusiform gyrus | -36 | -58 | -13 | 7.59 |
|  |  |  |  | L middle occipital gyrus | -42 | -82 | -1 | 7.54 |
|  |  |  |  | L calcarine cortex | 0 | -88 | -4 | 7.53 |
|  |  | 1819 | <0.001 | R inferior frontal gyrus, orbital part | 45 | 32 | -4 | 6.78 |
|  |  |  |  | R inferior frontal gyrus, triangular part | 54 | 38 | 8 | 6.72 |
|  |  |  |  | R inferior frontal gyrus, triangular part | 48 | 38 | 14 | 6.64 |
|  |  |  |  | R inferior frontal gyrus, opercular part | 42 | 11 | 20 | 5.9 |
|  |  |  |  | R amygdala | 30 | 2 | -19 | 5.86 |
|  |  |  |  | R inferior frontal gyrus, orbital part | 42 | 41 | -13 | 5.76 |
|  |  |  |  | R precentral gyrus | 39 | 2 | 38 | 5.62 |
|  |  |  |  | R inferior frontal gyrus, triangular part | 42 | 26 | 17 | 5.61 |
|  |  |  |  | R superior temporal gyrus | 51 | -4 | -13 | 5.47 |
|  |  |  |  | R superior temporal pole | 45 | 20 | -22 | 5.41 |
|  |  |  |  | R inferior frontal gyrus, triangular part | 57 | 23 | 29 | 5.38 |
|  |  |  |  | R inferior frontal gyrus, triangular part | 57 | 23 | 17 | 5.3 |
|  |  |  |  | R middle temporal gyrus | 48 | -7 | -16 | 5.23 |
|  |  |  |  | R superior temporal pole | 39 | 20 | -28 | 5.09 |
|  |  |  |  | R superior temporal pole | 51 | 8 | -13 | 5.05 |
|  |  |  |  | brain stem | 6 | -34 | -7 | 4.97 |
|  |  | 903 | <0.001 | L inferior frontal gyrus, triangular part | -48 | 35 | 11 | 6.51 |
|  |  |  |  | L precentral gyrus | -36 | -1 | 32 | 5.06 |
|  |  |  |  | L inferior frontal gyrus, triangular part | -54 | 17 | 29 | 4.78 |
|  |  |  |  | L superior temporal pole | -45 | 17 | -22 | 4.69 |
|  |  |  |  | L middle temporal pole | -51 | -1 | -16 | 4.57 |
|  |  |  |  | L inferior frontal gyrus, triangular part | -48 | 20 | 23 | 4.48 |
|  |  |  |  | L inferior frontal gyrus, orbital part | -39 | 32 | -7 | 4.47 |
|  |  |  |  | L inferior frontal gyrus, orbital part | -42 | 20 | -13 | 4.45 |
|  |  |  |  | L inferior frontal gyrus, triangular part | -39 | 17 | 26 | 4.26 |
|  |  |  |  | L inferior frontal gyrus, orbital part | -51 | 41 | -7 | 4.11 |
|  |  |  |  | L inferior frontal gyrus, opercular part | -45 | 8 | 23 | 4.08 |
|  |  |  |  | L insula | -36 | 14 | -4 | 4.04 |
|  |  |  |  | L anterior insula | -24 | 20 | -4 | 3.94 |
|  |  |  |  | L middle frontal gyrus, orbital part | -45 | 53 | -4 | 3.8 |
|  |  |  |  | L inferior frontal gyrus, triangular part | -54 | 20 | 2 | 3.68 |
|  |  |  |  | L superior temporal pole | -51 | 5 | -22 | 3.54 |

*Note.* Cluster *p* (FWE): cluster level family-wise error corrected p-value; L: Left hemisphere; R: right hemisphere; MNI: coordinates in Montreal Neurological Institute (MNI) space, Peak t-value: peak test-statistic of the one-sample t-test.

**Supplementary Figure 1.** Increased brain activation at a nominally significant level to fearful faces: Morning start > Varying start (Study 2).


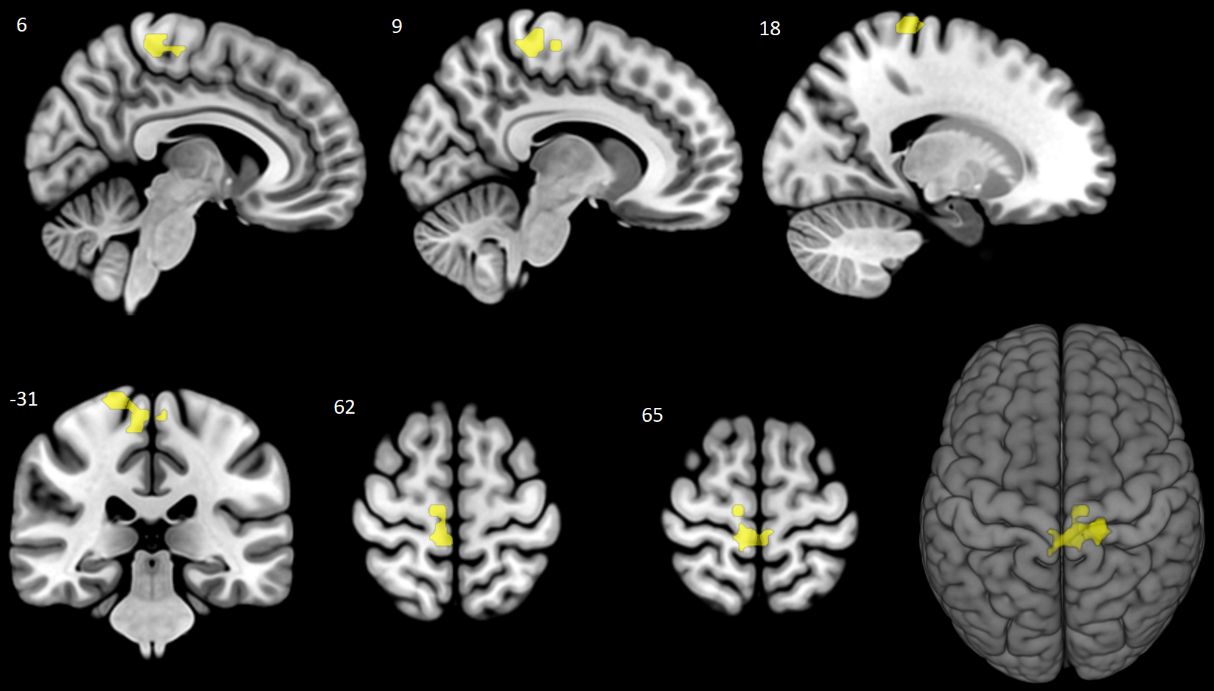


The Morning start M_circ_ subgroup showed increased brain activation compared to the Varying start subgroup in response to fearful faces. The nominally significantly activated cluster is shown with yellow color representing regions of bilateral paracentral lobule, right precentral gyrus and right supplementary motor area at a cluster level p_FWE_<0.05.
